# Human Papillomavirus Vaccination uptake and associated factors among schools girls aged between 9–14 years in Ethiopia: Performance Monitoring for Action (PMA-ET) 2023, multilevel analysis

**DOI:** 10.1371/journal.pone.0325557

**Published:** 2025-06-10

**Authors:** Ermias Bekele Enyew, Mulugeta Desalegn Kasaye, Shimels Derso Kebede, Mahider Shimelis Feyisa, Naol Gonfa Serbessa, Tsion Mulat Tebeje, Abiyu Abadi Tareke

**Affiliations:** 1 Department of Health Informatics, School of Public Health, College of Medicine and Health Sciences, Wollo University, Dessie, Ethiopia; 2 Department of Medical Laboratory, College of Health Science, Debre Tabor University, Debre Tabor, Ethiopia; 3 Department of Health Informatics, College of Health Science, Mattu University, Mattu, Ethiopia; 4 School of Public Health, College of Health Sciences and Medicine, Dilla University, Dilla, Ethiopia; 5 Zonal-Level COVAX and Routine Immunization Technical Assistance (TA) at West Gondar zonal health department, Gondar, Ethiopia; Greenebaum Cancer Center, Institute of Human Virology, University of Maryland School of Medicine, UNITED STATES OF AMERICA

## Abstract

**Background:**

Human papillomavirus (HPV) is one of the sexually transmitted diseases infections that causes cervical cancer, and it is the second-leading cause of infection-related cancer globally. HPV infection causes around 604,000 cervical cancer cases (342,000 deaths) globally each year. Therefore, this study aimed to assess Human Papillomavirus Vaccination uptake and associated factors among schools girls in Ethiopia.

**Method:**

Performance Monitoring for Action Ethiopia (PMA Ethiopia) is a survey project designed to generate data on various reproductive, maternal, and newborn health (RMNH) indicators that can inform national and regional governments. The prevalence of HPV vaccine uptake with a 95% Confidence Interval (CI) was reported and presented in a forest plot for East Africa Countries using STATA version 14.1. Intra-class Correlation Coefficient (ICC), Likelihood Ratio (LR) test, Median Odds Ratio (MOR), and deviance (−2LLR) values were used for model comparison and fitness. Adjusted Odds Ratios (AOR) with a 95% Confidence Interval (CI) and p-value ≤0.05 in the multilevel logistic model were used to declare significant factors associated with HPV vaccine uptake.

**Result:**

In Ethiopia, the prevalence of HPV vaccine uptake among schools girls was 30.82% (95% CI: 29.21, 32.45). In the multilevel logistic regression model, girls in age groups of 12–14 years were 2.44 [AOR = 2.44, 95% CI: 1.86–3.16] times more likely to take HPV vaccine as compared to girls aged 9–11 years. Similarly, girls who had received any health service and received sexual and reproductive health services had 7.75 [AOR = 7.75, 95% CI: 5.65–10.62], and 3.24 [AOR = 3.24, 95% CI: 2.33–4.51] were more likely to take HPV vaccine compared to their counterparts respectively.

**Conclusion:**

The study findings indicate that the proportion of girls reporting receipt of the HPV vaccine in this nationally representative survey is an alarmingly low 30.8%. The following critical factors have influenced this rate: age, access to sexual and reproductive health services, general health service utilization, and regional health disparity.

## Background

Human papillomavirus (HPV) is one of the sexually transmitted diseases infection that causes cervical cancer, and it is the second-leading cause of infection-related cancer globally [1,2]. There are numerous genotypes of HPV. HPV types 6 and 11 are responsible for 90% of genital warts, but HPV types 16 and 18 are classified as high-risk viruses, accounting for 70% of cervical cancer [3]. Persistent HPV infections with strains 16 and 18 cause 70% of cervical cancers and precancerous lesions [4]. Globally, HPV infection causes about 604,000 cases of cervical cancer annually, which leads to about 342,000 deaths. A significant amount of this burden falls on countries with low or middle incomes, where 90% of cases and 90% of deaths [5].

The frequency of cervical HPV infection varies around the world, but African women experience some of the highest rate [6]. Cervical cancer, caused by human papillomavirus (HPV), is the leading cause of cancer mortality among women in Sub-Saharan Africa (SSA) [7]. The estimated HPV prevalence in Sub-Saharan Africa is 24.4% [8]. A study conducted in Kenya revealed that HPV prevalence was high (42.3%), with approximately 46% of HPV-positive women carrying multiple kinds of infections. Another study conducted in Rwanda showed that HPV prevalence was 34%, being highest (54%) in women ≤19 years and decreasing to 20% at age ≥50 [9]. In a study done in some West African countries, the prevalence of HPV vaccine among women was 13.4% in Gambia [10], 33.2% in Benin [11], and 10.6 in Togo [12]. A small study in Gambella, Ethiopia, found that 48% of adolescent girls had received the HPV vaccine [13]. Similarly, in a study conducted in Bahirdar City among female preparatory school students, the proportion of human papillomavirus (HPV) vaccine uptake was 45.3% [14].

As of 2020, more than half of the WHO member countries have introduced HPV vaccination programs to meet the 2030 Sustainable Development Goal (SDG) elimination target of 90% [15]. The 2022 WHO position paper on HPV vaccinations urged that they be included in standard national immunization programs as a public health priority, including the goals related to immunization and their contribution to Primary Health Care (PHC) and Universal Health Coverage (UHC) [16,17]. Many countries, including Australia, the United Kingdom, and Canada, have developed public health programs for HPV vaccination, with school-based programs achieving high coverage in the target populations [18–20]. However, many sub-Saharan African countries that had been delayed in HPV vaccine introduction still have low coverage [21].

In the world poorest countries, where pre-screening and treatment are few, patients seek medical attention after a complication develops, and the majority lack a variety of prevention programs or services that primarily affect young, uneducated women [7]. Uptake of the HPV vaccine is known to be influenced by a number of factors, including the education level of girls, the household wealth index [14], household family size [22], the marital status of the mother [19], girls received any health service, girls received sexual and reproductive health service, girls mobile ownership [23], and girls working status [24].

In Ethiopia, the projected number of cervical cancer cases and deaths in 2018 is 6294 and 4884, respectively [23]. In the country, cervical cancer is the second highest cause of female cancer in women aged 15–44 years [25]. Ethiopia has around 29 million women aged 15 and older who are at risk of having cervical cancer [26]. Even though 20 million Ethiopian women were eligible for cervical screening, less than 1% were screened [27]. Vaccinating primary school-age girls is the most cost-effective public health intervention against cervical cancer since the vaccination targets girls who have not made their sexual debut [28].

According to the Ethiopian Ministry of Health (MoH), girls between the ages of 9 and 14 should receive the HPV vaccine before they start having sex and are exposed to the virus that can induce cervical alterations that can result in cancer [29]. The HPV vaccine was made available to 14-year-old females in Ethiopia in December 2018. However, because of a worldwide scarcity of HPV vaccines, the nation is only launching the vaccination in one age group (girls aged 14) in the first year. Depending on the vaccines availability worldwide, it plans to roll out the introduction to other age groups in the second year and beyond [30]. HPV vaccination is more challenging than other health campaigns because the targets are 9-to 14-year-old girls, decisions are typically made by their parents, and the information may not be credible [31]. Though Ethiopia has set guidelines to reach global standards, the life-course approach to cervical cancer prevention is in its early stages [32]. The WHO Strategic Advisory Group of Experts on Immunization (SAGE) has recommended that Ethiopia switch to a single-dose HPV vaccination in August 2023 because of the programmatic benefits and similar protection duration and efficacy to the two-dose schedule [33]. Despite the global immunization drive to prevent HPV-related morbidity, HPV vaccination uptake remains low in Ethiopia. Therefore, an evidence-based study on the HPV vaccine and associated factors is a cornerstone for tracking the progress of the program to eliminate diseases caused by HPV and achieve the stated goal. Thus, this research aimed to assess Human Papillomavirus Vaccination uptake and associated factors among school girls in Ethiopia.

## Method and materials

### Study design and study period

The study represents secondary data from the Performance Monitoring for Action Ethiopia survey. Performance Monitoring for Action Ethiopia (PMA Ethiopia) is a survey project designed to generate data on various reproductive, maternal, and newborn health (RMNH) indicators that can inform national and regional governments. The project conducted cross-sectional and cohort surveys to fill a data gap, collecting information not currently measured by other large-scale surveys. Its focus is measuring the comprehensiveness of RMNH care services and the barriers and facilitators to such care. The period of this cross-sectional study was November 2023 – January 2024 [34].

### Study area

Ethiopia was the site of the investigation. Ethiopia is situated in the Horn of Africa. It consists of two city administrative areas (Addis Ababa and Dire-Dawa) and twelve regional states (Tigray, Afar, Amhara, Oromia, Somali, Benishangul-Gumuz, Southern Nations, Nationalities, and People’s Region (SNNP), Gambella, Harari, Sidama, Southwest Ethiopia, and Central Ethiopia.

### Study population

All Ethiopian girls in the age range [9–14] included in the survey comprised the study population.

### Sampling technique and Sample size

The two-stage cluster design employed in the PMAET 2023 survey included regions and urban-rural areas as strata. A total of 280 Enumeration Areas (EAs) were chosen at random from the master sample frame, and 35 households were chosen from each EA. All women aged 15–49 in the selected households are eligible for the cross-sectional survey [34]. From among 6712 included in the broader survey, a total of 2963 girls met the inclusion criteria and had complete responses.

### Variable of study

#### Outcome variable.

The outcome variable was “Has received HPV vaccination?” This binary response variable indicated the uptake of the HPV vaccine. Girls who had received the HPV vaccination were categorized as having an uptake, coded as 1. While those who had not received the vaccine were coded as 0, indicating “not uptake HPV vaccinated.”

#### Independent variable.

**Individual-level factors:** respondents’ age (categorized as 9–11 and 13–14 years), girls’ education status, Household wealth index, household family size, maternal marital status, girls’ receipt of any health service, girls’ receipt of sexual and reproductive health service, girls’ mobile ownership, and girls’ working status were included as individual-level factors.

**Community-level factors**: a place of residency (rural, urban), community-level girl received any health service (low level, and high level), community-level girls received sexual and reproductive health service (low and high level), and regions were included under community-level factors.

### Operational definition

**Community level received any health service**: Number of girls who had received any health service. High levels of any health service received were defined as those who fall at or above the median value of the variables. Low levels of any health service received were defined as those that fall below the median value of the variables. Since the normalcy test of community-level poverty was skewed (the Jarque-Bera test’s p-value was less than 0.05, indicating a skewed distribution), the median was utilized as the cutpoint.

**Community-level sexual and reproductive health services received** a percentage of girls who had received sexual and reproductive health services. We classified this community-level factor in a manner comparable to that of any health service received.

### Data collection procedure

Three separate research activities comprise PMA-Ethiopia, a five-year (2019–2023) project executed in collaboration with Addis Ababa University, Johns Hopkins University, and the Federal Ministry of Health. These include yearly cross-sectional surveys of women aged 15–49, longitudinal surveys of women who are pregnant or have recently given birth, and annual service delivery point surveys of health facilities [34]. The Johns Hopkins School of Public Health received an online application through https://www.pmadata.org/data/available-datasets/request-accessdatasets, which was utilized to retrieve the PMA-Ethiopia datasets.

### Data management and analysis

Data were edited, coded, cleaned, and analyzed using STATA software version 14. STATA software was developed by the Computing Resource Center in California, and the first version was released in 1985 [35]. Descriptive statistics were employed using frequencies and percentages. The prevalence of HPV vaccine uptake with a 95% Confidence Interval (CI) was reported and presented in a forest plot for the Ethiopia region.

### Multilevel analysis

The PMA-ET data exhibits a hierarchical structure, where girls are nested within households, and households are further nested within clusters. This nesting can lead to intra-cluster correlation, meaning that girls within the same cluster may be more similar to one another than to those in different clusters. Consequently, using standard statistical models may underestimate the standard errors of effect sizes, which can distort the assessment of the null hypothesis and increase the risk of Type I errors. It suggests that advanced models have to be used to account for between-cluster variability. Therefore, the outcome variable was binary. In the multilevel logistic regression model, we ran four models to estimate both fixed effects of the individual and community-level factors and random intercept of between-cluster variation. The first null or unconditional model contained no predictor variable used to decompose the amount of variance between cluster levels. The second model consisted of only individual-level factors, whereas the third model had only community-level variables. The final model controlled both individual and community factors (full model).

### Intra-class correlation coefficient (ICC) and median odds ratio (MOR)

The Intra-Class Correlation (ICC) was used to express the random effects, or the amount of community variation, which are measures of variation in HPV vaccine uptake among communities or clusters. To determine whether there was variability or a clustering effect, the median odds ratio (MOR) was provided. When two clusters or EAs are randomly selected, it is defined as the median value of the odds ratio between the cluster with high odds of girls’ HPV vaccine uptake and the cluster with lower odds of girls’ HPV vaccine uptake [36].

The Likelihood Ratio (LR) test and deviance (−2LLR) values were utilized to evaluate model comparison and fitness since the models were nested [37]. Accordingly, a mixed-effect logistic regression model (fixed and random effect) was selected as the best-fitted model since it had the highest LLR and lowest deviance value, Akaike’s information criterion (AIC), and Bayesian information criterion (BIC). Variables with p-value < 0.2 in the bi-variable analysis were considered in the multivariable logistic regression model. Adjusted Odds Ratios (AOR) with a 95% Confidence Interval (CI) and p-value <0.05 in the multilevel logistic model were used to declare significant factors associated with HPV vaccine uptake.

### Ethics approval and consent to participate

There is no information gathering from subjects in this study. This specific study did not require participant permission or ethical approval. Given that, PMA-Ethiopia records provide the basis for the study’s secondary data analysis. The Johns Hopkins University Bloomberg School of Public Health (JHSPH) Institutional Review Board and Addis Ababa University College of Health Sciences (AAU/CHS) have granted ethical approval to PMA Ethiopia. Institutional Review Board and Addis Ababa University College of Health Sciences (AAU/CHS) approved the verbal consent. Written consent is not necessary when data collection involves non-invasive methods (such as collecting biospecimens) or in low-literacy areas, according to this guidance and the IRB’s records. Institutional review boards at JHBSPH and AAU approved the methods used for data collection [34]. We confirm all methods were carried out according to the relevant guidelines and regulations. The authors do not have access to any information related to personal identifiers during the data collection process.

## Results

### Socio-demographics characteristics of the respondents

A total of 2,963 people participated in the study. Most of the females were in the 12- to 14-year-old age range. In regards to residency and educational attainment, 2,565 (86.6%) were enrolled in primary school, while 2,216 (74.8%) lived in rural areas. The percentage of girls who had not received sexual and reproductive services was about 20%, while 1,215 (51.8%) had received any health services. (Table 1).

**Table 1:** Sociodemographic characteristics of the respondents, PMA-ET, 2023 (n = 2,963).

Variables	Frequency	Percent
**Age (in years)**		
9–11	1,337	45.12
12–14	1,626	54.88
**Educational status**		
No education	366	12.3
Primary education	2,565	86.6
Secondary education	32	1.1
**Household Wealth index**		
Poor	1,477	49.8
Middle	656	22.2
Richer	829	28.0
**Maternal Marital Status**		
Single	2,491	84.1
Married	472	15.9
**Household Family size**		
1–4 children	611	20.6
5–9 children	2,182	73.6
10+	170	5.7
**Working status**		
No	2,343	79.4
No	607	20.6
**Mobile ownership**		
No	2,838	95.8
Yes	125	4.2
**Place of residence**		
Urban	747	25.2
Rural	2,216	74.8
**Received sexual and reproductive health service**		
No	2,379	80.3
Yes	584	19.7
**Received any health service**		
No	1,132	48.2
Yes	1,215	51.8
**Community level Received sexual and reproductive health service**		
Low level	1,444	48.9
High level	1,506	51.1
**Community level Received any health service**		
Low level	1,504	51.3
High	1,426	48.7
**Region**		
Tigray	327	11.1
Afar	108	3.6
Amhara	405	13.7
Oromia	488	16.5
Somali	263	8.9
Benishangul-Gumuz	39	1.3
SNNP	414	14.0
Gambella	157	5.3
Harari	20	0.7
Addis Ababa	116	3.9
Dire Dawa	33	1.1
Sidama	198	6.7
South West Ethiopia	236	8.0
Central Ethiopia	146	4.9

### Prevalence of HPV uptake in Ethiopia

**Fig 1:**
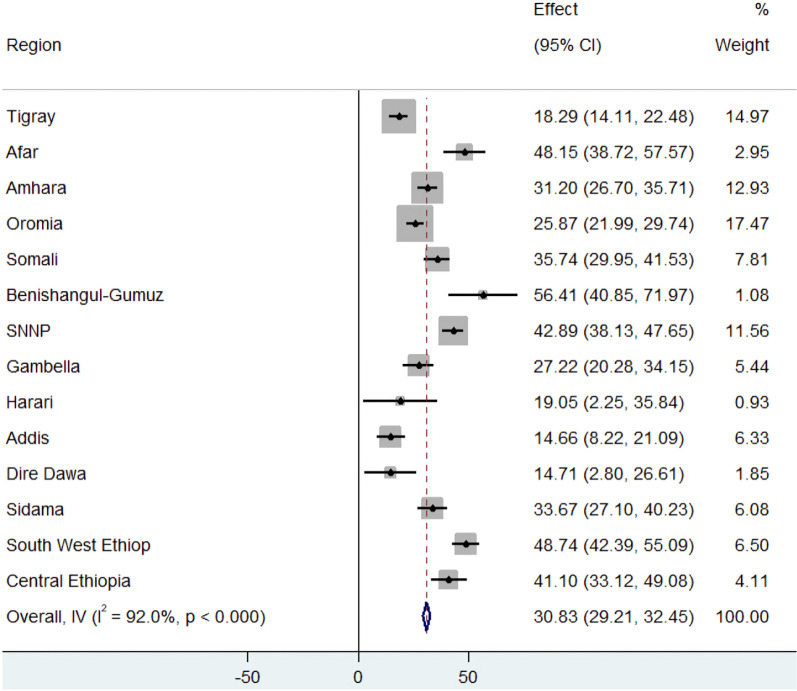
Prevalence of HPV vaccine uptake among girls by administrative region in Ethiopia, PMA-ET 2023.

In Ethiopia, the prevalence of HPV vaccine uptake was 30.82% (95% CI: 29.21, 32.45). There were regional differences: HPV vaccine uptake was most common in the Afar, Benishangul-Gumuz, southwest Ethiopia, central Ethiopia, and SNNP regions, while it was least prevalent in Addis Ababa and the Dire Dawa administrative zone of Ethiopia. (Fig 1). In a forest plot, the box shapes around each data point represent the effect estimates for each region.

### Multilevel logistic regression analysis

#### Factors associated with Human Papillomavirus Vaccination uptake in Ethiopia.

Multivariable multilevel logistic regression analysis revealed that, at the individual level, older age and having received any health service or sexual and reproductive health service and, at the community level, these factors included living in Afar, Somali, SNNP, and South-West Ethiopia were positively associated with vaccine uptake. The analysis supporting these findings is detailed below. Table 2 shows the random effect model. In model I, the ICC indicated that 41.3% of the total variability for HPV vaccine uptake was due to differences between clusters while the remaining unexplained 58.7% of the total variability of HPV vaccine uptake was attributable to individual differences. Additionally, the model I MOR of 3.92 (95% CI: 3.34, 4.60) showed that there was a difference in the uptake of the HPV vaccine between clusters. If we randomly selected two girls from different groups, the girls from the high cluster would be 3.92 times more likely to have HPV vaccine uptake than girls from the low cluster. The proportional change in variance (PCV) in this model was 56.3%, which showed that both community and individual-level variables explained 56.3% of community variance observed in the final model. The multilevel logistic regression model IV was the best-fitted model because it had the lowest values of AIC and BIC, the highest Log-likelihood Ratio (LLR), and the lowest deviation since the models were nested in the random effect.

**Table 2:** Model comparison and model fitness for multilevel logistic regression analysis.

Parameters	Model I	Model II	Model III	Model IV
**Random effect**				
Community variance	2.31[1.68,3.18]	1.91[1.44,2.55]	1.55[1.08,2.21]	**1.01[0.73,1.41]**
ICC%	41.3%	36.84%	32.0%	**23.6%**
MOR	3.92[3.34,4.60]	3.56[3.09,4.12]	3.21[2.68,3.83]	**2.59[2.20, 3.06]**
PCV%	1	17.3	32.9	**56.3**
**Model comparison**				
AIC	3311	3233	2212	**2327**
BIC	3323	3340	2293	**2185**
LLR	−1653	−1598	−1092	**−1062**
Deviance	3306	3196	2184	**2124**

NB: **AIC**: Akaike’s information criterion, **BIC**: Bayesian information criterion, **LLR**: Log likelihood Ratio, **MOR**: Median Odd Ratio, **ICC**: Intra-class Correlation Coefficient and **PCV** Proportional Change in Variance

### The fixed effects analysis result

In the multivariable mixed effect (individual and community-level factors) binary logistic regression analysis, age, girls who had received sexual and reproductive health services, and any health services, region were significant determinants of HPV vaccine uptake in Ethiopia. (Table 3).

**Table 3:** Multivariable multilevel logistic regression analysis of individual and community-level factors associated with HPV vaccination uptake.

Characteristics	Model I (95%CI AOR)	Model II (95%CI AOR)	Model III (95%CI AOR)	Model IV (95%CI AOR)
**Age** (**in year)**				
9–11		1		1
12–14		2.39[1.83,3.14]***		2.44[1.86,3.16]***
**Education status**				
No education		1		1
Primary		1.06[0.21,5.31]		0.95[0.18,4.08]
Secondary		1.58[0.22,11.02]		1.32[0.18,9.32]
**Maternal marital status**				
Single		1		1
Married		0.48[0.32,1.71]		0.43[0.31,1.91]
**Household wealth status**				
Poor		1		1
Middle		1.26[0.88,1.79]		1.17[0.82,1.66]
Rich		1.42[0.97,2.09]		1.51[0.98,2.33]
**Family size**				
0–4		1		1
5–9		1.22[0.90,1.66]		1.17[0.87,1.59]
Above 10		0.89[0.47,1.67]		0.80[0.43,1.51]
**Mobile ownership**				
No		1		1
Yes		1.26[0.67,2.34]		1.31[0.70,2.43]
**Received sexual and reproductive health service**				
No		1		1
Yes		3.31[2.40,4.56]***		3.24[2.33,4.51]***
**Received any health service**				
No		1		1
Yes		8.10[5.97,10.98]**		7.75[5.65,10.62]***
**Working status**				
No		1		1
Yes		1.29[0.86,1.93]		1.25[0.84,1.85]
**Community level factors**				
**Place of residency**				
Urban			1	1
Rural			1.78[0.54,1.11]	0.70[0.41,1.18]
**Received sexual and reproductive health service**				
Low			1.	1
High			2.43[1.72,3.43]***	1.19[0.75,1.87]
**Received any health service**				
Low			1.00	1.
High			2.48[1.76,3.49]**	0.97[0.61,1.52]
**Region**				
Tigray			1	1
Afar			2.58[1.02,6.54]	2.34[1.98,4.47]***
Amhara			1.70[0.88,3.29]	1.49[0.66,3.38]
Oromia			1.11[0.57,2.13]	1.08[0.48,2.44]
Somali			3.22[1.43,7.24]	2.94[1.93,4.20]**
Benishangul-Gumuz			3.29[0.93,11.67]	3.13[0.66,14.82]
SNNP			3.09[1.57,6.08]**	4.02[1.72,9.39]**
Gambella			1.40[0.59,3.35]	1.69[0.58,4.91]
Harari			1.05[0.15,6.99]	1.33[0.09,3.24]
Addis Ababa			0.53[0.20,1.36]	0.31[0.05,1.00]
Dire Dawa			0.64[0.12,3.23]	0.47[0.06,3.26]
Sidama			1.37[0.60,3.13]	1.22[0.43,3.39]
South West Ethiopia			2.99[1.39,6.44]	3.46[1.69,8.72]**
Central Ethiopia			1.36[0.47,3.90]	1.20[0.32,4.43]

*(P < 0.05), ** (P < 0.01), ***(P < 0.001)

**Model I:** without any independent variable

**Model II:** only individual-level factors

**Model III:** only community level factors

**Model IV:** both individual and community-level factors

**N.B:** All the different models were presented in separate columns to conserve space and reduce the overall size of the table

In this study, girls in age groups of 12–14 years were 2.44 [AOR = 2.44, 95% CI: 1.86–3.16] times more likely to take the HPV vaccine as compared to girls aged 9–11 years. Similarly, girls who had received any health service and received sexual and reproductive health services had 7.75 times [AOR = 7.75, 95% CI: 5.65–10.62], and 3.24 [AOR = 3.24, 95% CI: 2.33–4.51] were more likely to take HPV vaccine compared to their counterparts respectively.

The finding revealed that community-level factors were significantly associated with HPV vaccine uptake. Girls in Afar, Somali, SNNP, and South-west Ethiopia had 2.34 times [AOR = 2.34, 95% CI: 1.98–4.47], 2.94 [AOR = 2.94, 95% CI: 1.93–4.20], 4.02 [AOR = 4.02, 95% CI: 1.72–9.39], and 3.46 times [AOR = 3.46, 95% CI: 1.69–8.72] were higher the odd of HPV vaccine uptake compared to girls in Tigray, respectively. (Table 3).

## Discussion

This study aimed to assess the prevalence of HPV vaccine uptake among girls aged between 9–14 years in Ethiopia based on the most recent PMA data. Ethiopia’s ambitious goal to vaccinate over 7 million girls against the human papillomavirus (HPV) is crucial in the fight against cervical cancer, which poses a significant health risk to women in the country. As of April 2024, more than 6.3 million girls have received at least one dose of the vaccine, reflecting considerable progress in vaccination efforts [29]. The study reported a prevalence of HPV vaccine uptake at 30.8%, highlighting a significant discrepancy compared to the 89.9% two-dose coverage rate recorded in 2023 [38]. The observed variation in vaccination rates following the recent introduction of the HPV vaccination program in Ethiopia may be a lack of knowledge about one’s own risk of developing cervical cancer [39].

Similarly, this finding is lower than a study done in Ethiopia, the uptake of HPV vaccination among female students in Gambella Town (48.0%) [13], and Nekemte City (61.2%) [23], 44.4% in Ambo [40], 66.5% in Minjar Shenkora in North Shoa [28], 50.4% in Arba Minch [41] and 45.3% in Bahir Dar City [14]. A possible explanation might be the difference in the study setting. Adolescents who visited the school were recruited for earlier studies at the facility. These groups typically have greater access to health information and immunization information [42].

In addition, the finding is lower than a study done in Brazil [43], in Uganda (44.6%) [44], in Kibaha Town Council (47.92%) [45]. This disparity could be due to several factors, including differences in socioeconomic level between nations, access to the HPV vaccine, commitment to expansion, and knowledge of the vaccine [46]. Furthermore, disparities may arise from sociocultural barriers to healthcare services, particularly adolescent healthcare services in developing nations [40].

In the multivariable mixed-effect binary logistic regression analysis, age, girls who had received sexual and reproductive health services, and any health services and region were significant determinants of HPV vaccine uptake in Ethiopia. Adolescent girls’ adoption of the Human Papillomavirus (HPV) vaccine is essential for preventing cervical cancer, especially for those between the ages of 9 and 14. The human papillomavirus (HPV) vaccination campaign in Ethiopia aims to reduce the prevalence of 9–14-year-old girls who get cervical cancer when it typically presents at an older age period than this. This finding revealed that girls’ age had a significant effect on HPV vaccine uptake. Girls aged 12–14 had higher odds of taking the HPV vaccine compared to those aged 9–11. This finding is supported by a study conducted in Uganda [22,47], Indonesian [48], Nigeria [49], a prior study done in Nekemte City, Western Ethiopia [23], and a similar finding conducted in Wolida, Northeast, Ethiopia [39]. The possible reason might be a positive association between vaccine uptake and receipt of other services highlighting the importance of linking HPV vaccination to other adolescent healthcare service delivery [50]. Furthermore, as children get closer to the age of sexual debut, parents may feel more pressure to protect them from HPV-related infections, which could further influence vaccine decisions [51].

This finding revealed that girls who had received sexual and reproductive health were strongly positively associated with HPV vaccine uptake. This finding is supported by the study conducted elsewhere [23,52,53]. Similarly, this study revealed that the uptake of any health services was significantly associated with HPV vaccine uptake. This finding is collaborated by studies conducted in Uganda [22], in South Asia [54], in Germany [55], and in Mettu Town, Ethiopia [24]. Students’ decision to receive the vaccination may influenced by their belief that doctors are reliable sources of health information. Additionally, the availability of awareness-raising activities at their school (even if they are sporadic or brief) helps them learn more about the advantages of the vaccine, which in turn encourages them to receive it [40]. Moreover, there is a statistically significant relationship between the HPV vaccine uptake and the residential area. Compared to girls in the Tigray region, girls in Southwest Ethiopia, Somalia, SNNP, and Afar were more likely to receive the HPV vaccine. A potential reason could be that those areas share comparable cultures, religions, and customs.

### Strength and limitation

The use of large sample sizes and nationally representative data is the main strength of this study. A causal association cannot be established using PMA data, just like other cross-sectional data. As a result, these restrictions must be considered when analyzing or interpreting findings from this research.

## Conclusions

The study findings indicate that the proportion of girls reporting receipt of the HPV vaccine in this nationally representative survey is an alarmingly low 30.8%. In multilevel logistic regression results, age, access to sexual and reproductive health services, general health service utilization, and regional health disparity were significant associated factors with HPV vaccine uptake. These results highlight the critical need for focused public health initiatives that can raise immunization rates. Raising awareness, facilitating access, and assuring the quality of healthcare services should be the main goals to enhance HPV vaccination coverage, particularly in low-uptake areas.

## Abbreviations

AIC: Akakian Information Criteria
AOR: Adjusted Odds Ratio
BIC: Bayesian information criterion
CI: Confidence Interval
ANC: Antenatal Care
EAs: Enumeration Areas
HPV: Human Papilloma Virus
PMAS: Performance Monitoring for Action Survey
LLR: Log-Likelihood Ratio
ICC: Intraclass Correlation
MOR: Median Odds Ratio
PCV: Percentage Change in Variance
SNNP: South Nations and Nationalities People.
